# The foxconn suicides and their media prominence: is the werther effect applicable in china?

**DOI:** 10.1186/1471-2458-11-841

**Published:** 2011-11-02

**Authors:** Qijin Cheng, Feng Chen, Paul SF Yip

**Affiliations:** 1 Department of Social Work and Social Administration, 13th Floor, KK Leung Building, The University of Hong Kong, Pokfulam, Hong Kong SAR, China; 2 School of Mathematics and Statistics, University of New South Wales, Sydney, Australia; 3 HKJC Centre for Suicide Research and Prevention, No. 2, University Drive, The University of Hong Kong, Hong Kong SAR, China

## Abstract

**Background:**

Media reporting of suicide and its relationship with actual suicide has rarely been investigated in Mainland China. The "Foxconn suicides" is a description referring to a string of suicides/attempts during 2010, all of which were related to a giant electrical manufacturing company, Foxconn. This study aimed to examine the clustering and copycat effects of the Foxconn suicides, and to investigate temporal patterns in how they were reported by the media in Mainland China, Hong Kong (HK), and Taiwan (TW).

**Methods:**

Relevant articles were collected from representative newspapers published in three big cities in Mainland China (Beijing (BJ), Shenzhen (SZ), and Guangzhou (GZ)), HK, and TW, together with searching intensity data on the topic conducted using the Baidu search engine in Mainland China. The temporal clustering effects of the Foxconn suicides and their media prominence were assessed using the Kolmogorov-Smirnov test. The media reports of the Foxconn suicides' temporal patterns were explored using a nonparametric curve estimation method (that is, the local linear method). The potential mutual interactions between the Foxconn suicides and their media prominence were also examined, using logistic and Poisson regression methods.

**Results:**

The results support a temporal clustering effect for the Foxconn suicides. The BJ-based newspapers' reporting and the occurrence of a Foxconn suicide/attempt are each found to be associated with an elevated chance of a further Foxconn suicide 3 days later. The occurrence of a Foxconn suicide also immediately influenced the intensity of both Baidu searching and newspaper reporting. Regional diversity in suicide reporting tempo-patterns within Mainland China, and similarities between HK and TW, are also demonstrated.

**Conclusions:**

The Foxconn suicides were temporally clustered. Their occurrences were influenced by the reporting of BJ-based newspapers, and contagion within the company itself. Further suicide research and prevention work in China should consider its special media environment.

## Background

**Figure 1:**
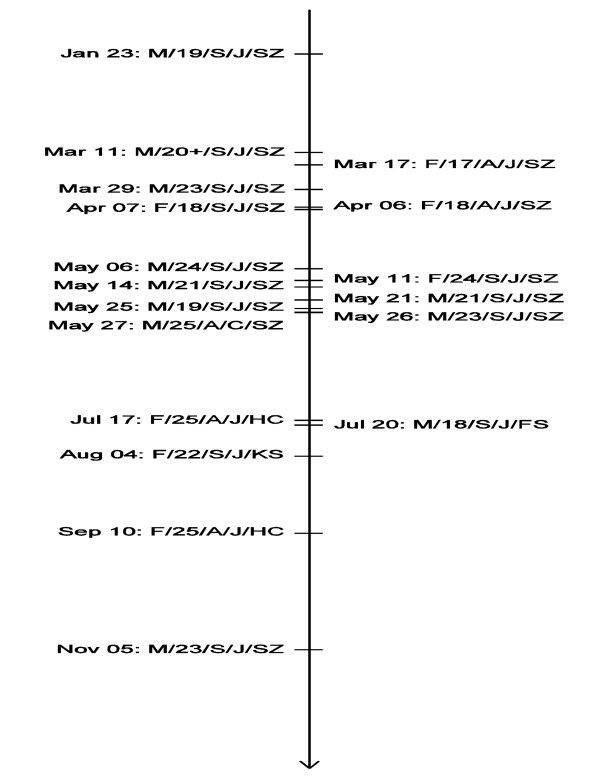
**Time distribution of the Foxconn suicides in 2010**. Each case is formatted as "date: gender (M = male, F = female)/age/suicidal behavior (S = completed suicide, A = suicide attempt)/suicide method (J = jumping, C = cutting)/location (SZ = Shenzhen, FS = Foshan, KS = Kunshan, HC = Hsinchu)".

The suicide rate in Mainland China has been estimated to be as high as 23 per 100, 000 individuals and the latest (2009) suicide rates in Hong Kong (HK) and Taiwan (TW), according to accurate census reports, were 13.8 and 17.6 per 100, 000, respectively [1-3]. Suicides in these three communities have accounted for more than a quarter of all such events worldwide [4]. However, the suicide problem in China was not recognized by the general public until the "Foxconn suicides." In this paper, this label is used to refer specifically to 13 completed and 5 attempted suicides in 2010, all of which were connected to a single company, Foxconn (see detailed case profile in Figure 1). The description was coined by the mass media and used thereafter to refer to the 18 cases.

Foxconn is a Taiwanese-owned company but most of its factories are in Mainland China. It manufactures and assembles consumer electronic products for companies like Apple, Nokia, Dell, and so on. The string of suicides/attempts attracted mass media concern about the suicide problem in modern China in all Chinese communities, and was even discussed by some international media, such as the New York Times and the Wall Street Journal [5,6].

During the development of the Foxconn suicides, two assertions were widely reported by the Chinese media without any empirical evidence being produced; 1) that the Foxconn suicides formed a cluster [7,8], and 2) that they were attributable to media reporting [8-10]. Given that this may be the first time the Chinese public has had the opportunity to recognize suicide as a public health problem, it is necessary to examine these assertions scientifically, to avoid people being influenced by wrong or misleading ideas. Therefore, we conducted an empirical study on the Foxconn suicides with the aim of examining the two propositions outlined above.

For the first assertion, given that the Foxconn suicides took place in different cities but were all relevant to the same company, we hypothesized that:

H1. There was a temporal clustering effect among the Foxconn suicides.

For the second assertion, it is known that the inappropriate reporting of suicides, especially among celebrities, is related to consequent macro-level increases in suicide rates. This is commonly referred to as the *Werther Effect *[11,12]. However, previous studies in this field have been done mostly in western countries or developed Chinese communities such as HK and TW [13,14]. The influence of the mass media on real suicides has not yet been empirically examined in Mainland China [15]. More importantly, previous studies often examine the relationship between media reporting and changes of suicide rates in the general population, rather than in terms of individual cases [11,12].

According to previous studies, suicide clusters can be divided into two groups: *point clusters*, which occur within a small community or institution, and *mass clusters*, which are found in the general population [16]. The Foxconn suicides were all related to one company so, if they were a cluster at all, should be placed in the former category. A point cluster can be caused by social learning within a local network or by the tendency for similar individuals to share similar risk factors and commit suicide in association with one another. However, the relationship between a point cluster and prominent mass media reporting of suicides is still unproven [16,17]. Therefore, we hypothesize that:

H2. The mass media focus on the Foxconn suicides did not significantly influence their occurrences; and H3. Previous Foxconn suicides significantly influenced later occurrences.

As well as testing these three hypotheses, we also used the Foxconn suicides to investigate one further research question. We compared the prominent patterns of media reporting of the same cases in Mainland China, HK, and TW. The Mainland Chinese mass media's style of reporting suicide has rarely been examined. One recent study demonstrated different styles between Guangzhou (GZ: a city in Mainland China), HK, and TW. This encouraged us to expand our investigation to more regions in Mainland China.

In this study, we define "mass media" to include not only traditional media (that is, newspapers) but also new media, specifically online search engines. Internet penetration is over 70% in HK and TW and over 55% in relatively developed Mainland regions such as Beijing (BJ) and Guangdong Province [18-20]. With the widespread use of the Internet, more and more media researchers have turned to its use by the public for information seeking as a measure of a news topic's prominence [21]. However, as far as we know, no study has yet examined the influence of such online searching on suicide occurrences.

We hope our study can narrow the huge margin on media and suicide research in Mainland China and also explore the prominence of the relationship between the media (including both traditional media and the Internet) and individual suicide cases. In addition, previous studies of media and suicide have often been concerned with single rather than multiple cases, and with the deaths of celebrities rather than ordinary people [11,22]. Our study on the Foxconn suicides, covering as it does the suicides or attempts of 18 ordinary people, will enrich the literature on media and suicide.

## Methods

### Data collection

The 18 suicide cases, and detailed, relevant information about them, were identified by reviewing media reports. Both the company itself, and the Chinese government, held general media reporting of the Foxconn suicides responsible for further occurrences [9,10]. Therefore, we tried to include a wide range of Chinese newspapers in our examination. Not only Mainland Chinese newspapers, but also those published in HK and TW were included to examine the potential influence of media reporting. Within Mainland China, not only those newspapers distributed in Shenzhen (where most of the Foxconn suicides occurred) were included, but also those available in the larger Chinese cities and whose content was accessible online. National daily newspapers in Mainland China were not included because they might not be comparable with their HK and TW compatriots due to the huge differences in population and readership.

Specifically, three big cities with mature and active media markets in Mainland China, namely Shenzhen (SZ), GZ, and BJ, were selected to compare with HK and TW. SZ, a special economic zone in Guangdong Province which borders on HK, is the city in which most of the Foxconn suicides occurred. GZ is the capital city of Guangdong Province and is about an hour by train from SZ. As a provincial capital city, newspapers in GZ cover stories from around the province and have correspondents in major Guangdong cities, including SZ. BJ is the capital and the political, cultural, and media center of Mainland China. Its local daily newspapers, although they mainly cover local news, are also concerned with national events.

Following previous studies on HK and TW media, four popular daily newspapers with significant circulation figures and a wide range of reader profiles in each region were included in this study (see Table 1 for details) [23,24]. All 18 suicides/attempts were committed by jumping from a height, other than one attempt where the individual concerned cut his wrists. Accordingly, "Foxconn" and "suicide" or "jump" or "fall" or "cut" in Chinese were entered into the WiseNews database to search on the titles and content of all 20 newspapers included in the sample. The time period for the search was set as the full year 2010, from Jan 1 to Dec 31. A total of 1282 articles returned from the search, including news articles and commentaries, were then collected. After excluding articles that were not actually relevant to the Foxconn suicides, 1279 remained for content analysis and their full text versions were downloaded. Some basic characteristics, including the newspaper's name, circulation region, and the article's publication date were collected directly from WiseNews. To measure the prominence of the Foxconn suicides in each newspaper, the word count and placement (that is, whether or not it appeared on the front page) were also collected for each article.

**Table 1:** Descriptions of the 20 selected newspapers and the clustering effects of their reporting intensities of the Foxconn suicides.

ID	Circulation region	Newspaper	Total amount of articles	P-value of the test of clustering effect
P1	TW	Apple Daily	89	0.00
P2	TW	Liberty Times	71	0.00
P3	TW	United Daily News	125	0.00
P4	TW	China Times	83	0.00
P5	HK	Apple Daily	109	0.00
P6	HK	Ming Pao	138	0.00
P7	HK	Sing Tao	128	0.00
P8	HK	Oriental Daily News	68	0.00
P9	GD	Southern Metropolis Daily	92	0.00
P10	GD	Nanfang Daily	40	0.00
P11	GZ	Yangcheng Evening News	50	0.00
P12	GZ	Guangzhou Daily	34	0.00
P13	SZ	Daily Sunshine	51	0.00
P14	SZ	Shenzhen Economics Daily	21	0.02
P15	SZ	Shenzhen Special Zone Daily	34	0.00
P16	SZ	Shenzhen Evening News	34	0.00
P17	BJ	Beijing News	42	0.00
P18	BJ	Beijing Youth Daily	37	0.00
P19	BJ	Beijing Evening News	19	0.22
P20	BJ	Fa Zhi Evening News	14	0.02

Baidu searching trends relative to the Foxconn suicides were also collected, as a measure of online user-generated prominence. While western studies often cite Google Trend data as an indicator of aggregated online attention, Baidu, a Chinese search engine company, has captured over 70% of the Mainland market [25]. It allows users to enquire how many searches have been done on a certain term or phrase on Baidu over a set period (URL: http://index.baidu.com), a function similar to Google Trend. Following Weeks and Southwell, we tried several different search queries relevant to the Foxconn suicides and noticed that *Fu Shi Kang Tiao Lou *("Foxconn jump" in Chinese) generated the highest overall search index rating compared to the other tested terms. Accordingly, this term was ultimately chosen for the data collection [21].

### Data analysis

The dates of the 18 suicides/attempts are shown in Figure 1, which immediately suggests a clustering phenomenon. To assess the statistical strength of the evidence, we assumed the Foxconn suicides to have occurred according to a Poisson process and hence tested the constancy of its intensity or its equivalent, the uniformity of the distribution of the event times of the Poisson process over the observation interval. Specifically, the Kolmogorov-Smirnov test was employed to test the null hypothesis that the time distribution of the Foxconn suicides over the year of 2010 was uniform against the two-sided alternative hypothesis that it was not. The null hypothesis is equivalent to a lack of a temporal clustering effect for the Foxconn suicides, and the alternative hypothesis to the presence of such an effect. If the null hypothesis falls to be rejected, we would then use the local linear method to estimate the time-varying intensity function, which would help interpret the clustering [26,27]. The potential clustering effects among the newspaper reporting and Baidu searching of the Foxconn suicides were also assessed using the Kolmogorov-Smirnov test, and the corresponding time-varying intensities estimated using the local linear method. At this point, it is important to clarify that the intensities of specific types of events (such as the Foxconn suicides, the newspaper reporting of them, or the resulting online searching) are the intensity functions of the respective Poisson processes used to model these specific types. So if *N*(*t*) denotes the cumulative number of a specific type of events from time 0 (the beginning of the year 2010) up to time *t*, then *N*(*t*) is assumed to be a Poisson process, and the intensity of the event at time *t *is defined as:

(1) $$\lambda \left(t\right)=\underset{\Delta t↓0}{\lim}\frac{E\left\{N\left(t+\Delta t\right)-N\left(t\right)\right\}}{\Delta t}.$$

**Figure 2:**
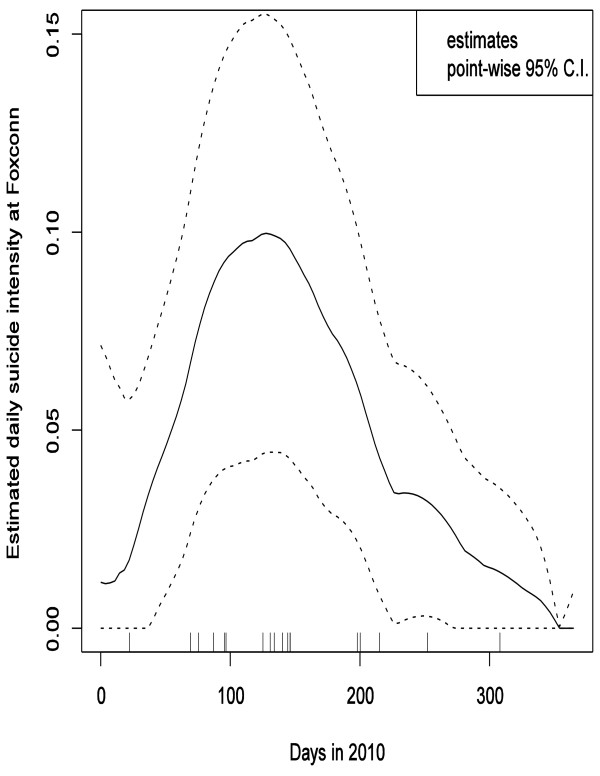
**Estimated daily suicide intensity at Foxconn in 2010 and its pointwise 95% confidence intervals**.

To explore the potential mutual influences between the media prominence and actual subsequent occurrences of the Foxconn suicides/attempts, we performed the logistic and Poisson regression analyses described below. Since the potential influence of media coverage on future suicides may not be immediate, but equally may not last very long [28], the time-lagged variables were set as regressors. As suggested by the time lag plots (Additional File 1), a lag range from 1 to 5 days was adopted. In Figure 2, the trend lines were obtained by the LOWESS scatterplot smoother [29].

To describe the models in more specific terms, let *x_t _*=(*x*_1*t*_, *x_2t_*, ..., *x_5t_*), *t *= 1, ..., 365, denote the series of vector-valued counts of interest, where *x*_1*t*_, *x_2t_*, and *x_3t _*denote the numbers of Foxconn suicide stories published by TW and HK papers, GZ and SZ papers, and BJ papers respectively; *x*_*4*t _denotes the amount of Baidu searching on the keyword; and *x_5t _*denotes the number of Foxconn suicides/attempts on day *t*. The variable *x_5t _*has values 0-1, and we modeled it using a Bernoulli distribution with probability of success *P_t _*which, after a logit transformation, depended linearly on the values of the series on the previous *q *days. More specifically, the model assumed that

(2) $$\begin{array}{c} x_{5t}\sim \text{Bernoulli}\left(p_{t}\right);\\ \text{logit}\left(p_{t}\right)=\log\frac{p_{t}}{1-p_{t}}=\beta_{0}+\overset{5}{\underset{i=1}{\sum }}\overset{q}{\underset{j=1}{\sum }}x_{i,t-j}\beta_{ji}, \end{array}$$

where *β*_0 _and *β_ji_*, *j *= 1, ..., *q*, *i *= 1, ..., 5, were model parameters. Since the variables *x*_1*t*_, ..., *x*_4*t*_, were low counts with zeros, a natural model to consider, when assessing the influence of other variables on a specific variable, was the Poisson regression model. Rather than using the ordinary case, a quasi-Poisson regression was adopted to account for the possible over-dispersion phenomenon and hence reduce the chance of detecting falsely-significant variables. More specifically, the mean of the response variable *μ_it _= E*[*x_it_*], *i *= 1, ..., 4, after the log transformation, was assumed to be a linear function of the values of the time series on the past *q *days, and the variance of the response was assumed to be equal to that expected for a Poisson distribution up to a positive scale parameter *ϕ*, known as the *dispersion parameter*. That is,

(3) $$\begin{array}{c} \log\left(\mu_{kt}\right)=\beta_{0}+\overset{5}{\underset{i=1}{\sum }}\overset{q}{\underset{j=1}{\sum }}x_{i,t-j}\beta_{ji},\;\\ v_{kt}=\text{var}\;\left(x_{kt}\right)=\varphi \mu_{kt},\;\;\;k=1,\ldots ,\;4, \end{array}$$

where the parameters *β*_0_, *β_ji_, j = *1, ..., *q*, *i *= 1, ..., 5, and *ϕ *depend on which variable acts as the response variable.

The logistic (2) and log-linear (3) models were all fitted using the General Linear Model (GLM) function of the statistical package R [30]. When fitting these regression models, we only used data from day 14 onward. This was because there were no suicides/attempts during the first 14 days of 2010. There were two newspaper articles over this period about one suicide at a Foxconn factory in July 2009. Since that case was otherwise not included in the Foxconn suicides by the mass media, it was also excluded from this study.

## Results

### Clustering effect

The *P *value of the Kolmogorov-Smirnov test of uniformity of the Foxconn suicides' time distribution was 0.002 < 0.05, so the null hypothesis (no clustering of suicide times) can be firmly rejected in favor of the alternative hypothesis that there was a temporal clustering of the Foxconn suicides. Figure 2 presents the local linear estimates of the suicide intensity together with point wise 95% confidence intervals using the Epanechnikov kernel and the rule of thumb bandwidth calculated from the data [27]. Figure 2 shows a clear peak in the suicide intensity occurring roughly between day 70 to day 150, or mid-March to May 2010. This corresponds well with the clustering of most of the Foxconn suicides in this period. Therefore, H1 is supported.

### Prominence tempo-pattern

**Figure 3:**
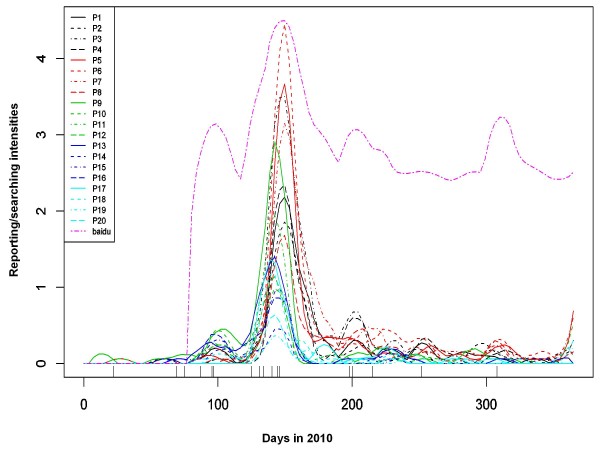
**Estimated newspaper reporting intensities and online searching intensity for the Foxconn suicides in 2010**. The descriptions of P1-20 can be found in Table 1. The Baidu searching intensity curves on a log transformed scale.

**Figure 4:**
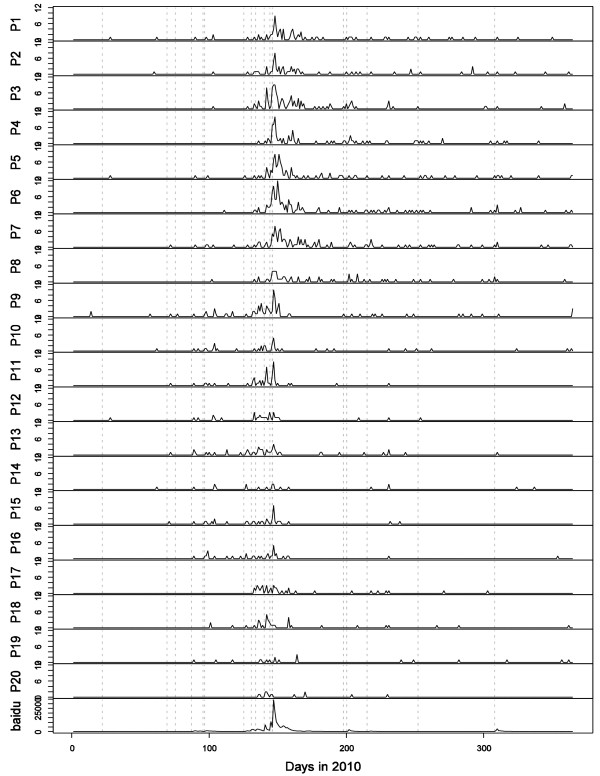
**Daily amount of newspaper reporting and online searching on the Foxconn suicides in 2010**. The descriptions of P1-20 can be found in Table 1. The broken grey lines indicate occurrences of the Foxconn suicides.

Figure 3 shows the estimated reporting intensities of the 20 selected newspapers and the estimated searching intensity on Baidu. These tend to suggest that while different newspapers or media types had clearly different scale of reporting/search intensities, their tempo-patterns were more or less consistent, with all showing clear peaks around clusters of suicides or isolated cases. A closer inspection shows that the peaks of the newspapers' reporting intensities in the same region were very well aligned, but those of newspapers from different regions tended to differ. This phenomenon can also be seen from Figure 4, which shows the daily amounts of news reporting and online searching running in parallel.

According to their reporting patterns, the 20 newspapers can be divided into three categories: HK- & TW-based, GZ- & SZ-based, and BJ-based. The time period can also be divided into three phases. From January to mid-May 2010, the GZ- & SZ-based papers' reporting intensities reflected the time distribution of the Foxconn suicides, whereas those of other newspapers under-reflected it. From mid-May to early June 2010, all the newspapers' reporting intensities increased strongly, reflecting the peak of the Foxconn suicides' intensity. After that, the HK- & TW-based papers' reporting intensities continued to mirror the pattern of the Foxconn suicides, whereas those of the Mainland Chinese newspapers all dropped to a much lower level. During the whole of 2010, the BJ-based papers showed a very low reporting intensity for the Foxconn suicides. No significant searching for the topic on Baidu took place until 29 March 2010, the date of the fourth Foxconn suicide. However, from 30 March 2010 onwards, the temporal pattern of Baidu searching intensity was fairly consistent with that of the reporting intensity of the newspapers, especially those in TW and HK.

### Interactions between the Foxconn suicides and their media prominence

The results of the regression analysis are shown in Table 2. Of all the 20 newspapers and one search engine studied, only the coverage in the BJ-based newspapers significantly contributed to the occurrences of Foxconn suicides 3 days later. A Foxconn suicide/attempt on a certain day also significantly contributed to a subsequent occurrence 3 days later. Therefore, H3, but not H2, is supported.

**Table 2:** Interactions between the occurrences of the Foxconn suicides and their media prominence.

Independent variables	Estimate	Std. Error	**Est./S.E**.	P value	**Sig**.
*The Foxconn suicides' occurrences as dependent variable*					

(Intercept)	-3.11	0.37	-8.36	0.00	Y-
TWHK newspapers.lag1	-0.32	0.24	-1.32	0.19	
TWHK newspapers.lag2	0.10	0.20	0.47	0.64	
TWHK newspapers.lag3	-0.42	0.24	-1.74	0.08	
TWHK newspapers.lag4	-0.12	0.23	-0.53	0.60	
TWHK newspapers.lag5	-0.26	0.22	-1.18	0.24	
GZSZ newspapers.lag1	-0.27	0.26	-1.01	0.31	
GZSZ newspapers.lag2	0.30	0.20	1.47	0.14	
GZSZ newspapers.lag3	-0.06	0.20	-0.29	0.77	
GZSZ newspapers.lag4	0.01	0.20	0.03	0.98	
GZSZ newspapers.lag5	-0.01	0.18	-0.04	0.97	
BJ newspapers.lag1	-0.64	0.71	-0.90	0.37	
BJ newspapers.lag2	0.08	0.49	0.16	0.88	
BJ newspapers.lag3	1.13	0.53	2.14	0.03	Y+
BJ newspapers.lag4	0.41	0.66	0.63	0.53	
BJ newspapers.lag5	-0.57	0.70	-0.82	0.42	
Baidu.lag1	0.00	0.00	1.29	0.20	
Baidu.lag2	0.00	0.00	0.57	0.57	
Baidu.lag3	0.00	0.00	0.67	0.50	
Baidu.lag4	0.00	0.00	-0.11	0.91	
Baidu.lag5	0.00	0.00	0.64	0.52	
Foxconn Suicides' occurrences.lag1	1.26	1.02	1.23	0.22	
Foxconn Suicides' occurrences.lag2	-4.26	3.42	-1.25	0.21	
Foxconn Suicides' occurrences.lag3	2.31	1.10	2.11	0.04	Y+
Foxconn Suicides' occurrences.lag4	-2.13	2.36	-0.91	0.37	
Foxconn Suicides' occurrences.lag5	-0.14	1.60	-0.08	0.93	

*Hong Kong and Taiwan newspapers' daily reporting amount as dependent variable*					

Foxconn Suicides' occurrences.lag1	-1.38	0.58	-2.39	0.02	Y-
Foxconn Suicides' occurrences.lag2	1.27	0.29	4.32	0.00	Y+
Foxconn Suicides' occurrences.lag3	1.37	0.31	4.48	0.00	Y+
Foxconn Suicides' occurrences.lag4	0.76	0.44	1.74	0.08	
Foxconn Suicides' occurrences.lag5	0.29	0.42	0.69	0.49	

*Guangzhou and Shenzhen newspapers' daily reporting amount as dependent variable*					

Foxconn Suicides' occurrences.lag1	-0.13	0.51	-0.25	0.80	
Foxconn Suicides' occurrences.lag2	1.93	0.29	6.54	0.00	Y+
Foxconn Suicides' occurrences.lag3	1.32	0.35	3.73	0.00	Y+
Foxconn Suicides' occurrences.lag4	0.43	0.54	0.79	0.43	
Foxconn Suicides' occurrences.lag5	0.25	0.50	0.50	0.62	

*Beijing newspapers' daily reporting amount as dependent variable*					

Foxconn Suicides' occurrences.lag1	0.32	0.74	0.43	0.67	
Foxconn Suicides' occurrences.lag2	0.88	0.59	1.50	0.13	
Foxconn Suicides' occurrences.lag3	0.00	0.61	-0.01	0.99	
Foxconn Suicides' occurrences.lag4	1.45	0.65	2.25	0.02	Y+
Foxconn Suicides' occurrences.lag5	0.79	0.59	1.34	0.18	

*Baidu searching's daily searching amount as dependent variable*					

Foxconn Suicides' occurrences.lag1	0.93	0.20	4.60	0.00	Y+
Foxconn Suicides' occurrences.lag2	1.22	0.19	6.56	0.00	Y+
Foxconn Suicides' occurrences.lag3	0.78	0.23	3.42	0.00	Y+
Foxconn Suicides' occurrences.lag4	0.92	0.26	3.49	0.00	Y+
Foxconn Suicides' occurrences.lag5	0.47	0.24	1.96	0.05	

In addition, occurrences of Foxconn suicides had an immediate influence on the amount of Baidu searching at lag 1 and a continuous influence until lag 4. They had a relatively slower influence on the amount of GZ, SZ, HK, & TW newspapers reporting at lag 2 and 3, and the slowest influence on all the BJ-based newspapers at lag 4.

## Discussion

The study echoes previous work showing that a point cluster can be caused by a copycat effect within a small community or institution. The influence of previous Foxconn suicides on later such events can be attributed to interpersonal communication within the company. Foxconn provides workers with a huge campus containing dorms, canteens, and other facilities, which results in a close community [7]. In addition, mobile phone penetration is over 64% in Mainland China and almost 100% in Guangdong Province [31], which can lead to the fast spread of information within a group.

It is not surprising that most of the mass media prominence had little influence on the occurrences of Foxconn suicides. The Foxconn suicides were all committed by ordinary people, and the evidence suggests that media reporting of such suicides rarely leads to a copycat effect [22,32]. Strikingly, the coverage of the Foxconn suicides in the BJ-based newspapers did have a significant influence on future occurrences. More interestingly, the BJ-based newspapers actually showed the lowest interest in, and the slowest follow up of, the Foxconn suicides among all the media studied here. This somewhat unexpected phenomenon might be explained by the special nature of BJ newspapers. Unlike the HK and TW media, Mainland Chinese media are widely considered as the mouthpiece of the Chinese government. BJ newspapers, located as they are in the nation's capital, are often considered by the public to be the mouthpiece of both the local and central government. BJ newspapers' concern with the Foxconn suicides might have been perceived as an authoritative message from the Chinese government, which may unfortunately have encouraged some vulnerable individuals to commit suicide to attract public attention. If such an explanation is plausible, we should pay more attention to the special media environment in Mainland China when conducting future studies on media and suicide.

As part of the exploration carried out in this study, the online media's role in spreading the news of the Foxconn suicides is also noteworthy. Although the Foxconn suicides were associated with a subsequent increase in intensity for all the media coverage studied here, the impact of a Foxconn suicide on Baidu searching was the most immediate and longest lasting. This demonstrates the speed and duration of the response generated by online users' attention to the phenomenon.

### Public health implications

Of the two assertions reported by the media, the one that the Foxconn suicides formed a cluster is supported by our empirical study. However, the other one that the Foxconn suicides were attributable to media reporting is only supported under certain conditions. The finding that the reporting by the BJ newspapers, but not the newspapers in other regions, influenced further Foxconn suicides reminds us to be more careful to balance the competing concerns of press freedom and the minimization of harm. It is therefore necessary to report our findings to professionals and policymakers who are working on, or interested in, suicide prevention in China, as well as the general public.

Suicide prevention efforts in China should be pursued, including introducing media guidelines on reporting suicides. However, the current guidelines are based largely on western studies [33]. More China-specific studies on media and suicide are needed to optimize these guidelines for the special media environment in China. In addition, the diversity of media reporting patterns in Mainland China is noteworthy. Those who seek to cooperate with the media to promote suicide prevention in China should pay attention to these features and design a flexible working strategy. To promote and practice suicide prevention in the new media environment, they should also consider contributing more suicide prevention content to the Internet for online searchers.

## Limitations

The study also has some limitations, which call for further research. Additional studies seeking to explain why the BJ-based newspapers influenced the Foxconn suicides are required. In-depth content analysis and qualitative interviews with relevant journalists and Foxconn workers may be helpful in understanding this phenomenon. In addition, due to the lack of daily suicide rate data in Mainland China, this study has only examined the influence of media prominence on the 18 Foxconn suicides. Nevertheless, it has explored an alternative approach to studying the media's effect on copycat suicides in a country where census data on suicide is not available.

## Conclusions

This study, utilizing advanced statistical methods, supports the hypothesis that the Foxconn suicides formed a temporal cluster. Of the 20 newspapers and one online search engine studied here, only the BJ-based newspapers' reporting of the Foxconn suicides significantly influenced subsequent occurrences. At the same time, previous Foxconn suicides also significantly contributed to later ones, which suggest a social learning phenomenon at work within the company.

The dynamics of the media's influence on copycat suicide is complex and its investigation is just beginning. Our study is pioneering in its examination of the Mainland Chinese media's tempo-patterns of reporting on suicides and their relationship with a suicide cluster. It highlights more questions and possibilities for this field of study. The Mainland China media environment is special due to the Chinese government's censorship and the media's struggle with this in the context of the emerging market economy. Previous findings from western countries, and even other Chinese societies like HK and TW, may not be directly applicable to Mainland China. To work with the media to prevent suicide in China, the relevant professionals and policymakers need to pay attention to its special media environment and develop flexible strategies.

## Competing interests

The authors declare that they have no competing interests.

## Authors' contributions

The study was initiated by QC and PSFY. QC designed the study, then collected and coded the data. FC helped with the data analysis and processed the figures. PSFY supervised the study and acted as coordinator. All three authors participated in the explanation and discussion of the results. The submitted manuscript was drafted by QC and discussed and agreed upon by the other two authors. All authors read and approved the final manuscript.

## Pre-publication history

The pre-publication history for this paper can be accessed here:

http://www.biomedcentral.com/1471-2458/11/841/prepub

## Supplementary Material

Additional file 1 **Lag plots of the count time series with the occurrences of the Foxconn suicides as target variables**. The file contains a figure showing lag plots of the count time series with the occurrences of the Foxconn suicides as target variables. Figure legend: Suic = the Foxconn suicides, TWHK = Taiwan and Hong Kong newspapers' daily reporting amount, GDSZ = Guangzhou and Shenzhen newspapers' daily reporting amount, BJ = Beijing newspapers' dailiy reporting amount, Baidu = Baidu's daily searching amount. Click here for file
